# Carbamazepine reduces disease severity in a mouse model of metaphyseal chondrodysplasia type Schmid caused by a premature stop codon (Y632X) in the *Col10a1* gene

**DOI:** 10.1093/hmg/ddy253

**Published:** 2018-07-14

**Authors:** Mitra Forouhan, Stephan Sonntag, Raymond P Boot-Handford

**Affiliations:** 1 Wellcome Centre for Cell-Matrix Research, Faculty of Biology, Medicine and Health and Manchester Academic Health Science Centre, University of Manchester, Manchester, UK; 2 PolyGene AG, Riedmattstr. 9, CH-8153 Rümlang, Switzerland

## Abstract

Mutations, mostly in the region of the *COL10A1* gene encoding the C-terminal non-collagenous domain, cause the dwarfism metaphyseal chondrodysplasia type Schmid (MCDS). In most cases, the disease mechanism involves the misfolding of the mutant protein causing increased endoplasmic reticulum (ER) stress and an unfolded protein response (UPR). However, in an iliac crest biopsy, the *COL10A1* p.Y632X mutation was found to produce instability of the mutant mRNA such that little mutant protein may be produced. To investigate the disease mechanism further, a gene-targeted mouse model of the *Col10a1* p.Y632X mutation was generated. In this model, the mutant mRNA showed no instability, and in mice heterozygous for the mutation, mutant and wild-type mRNAs were present at equal concentrations. The protein was translated from the mutant allele and retained within the cell, triggering increased ER stress and a UPR. The mutation produced a relatively severe form of MCDS. Nevertheless, treatment of the mice with carbamazepine (CBZ), a drug which stimulates intracellular proteolysis and alleviates ER stress, effectively reduced the disease severity in this model of MCDS caused by a premature stop codon in the *Col10a1* gene. Specifically, the drug reduced ER stress in the growth plate, restored growth plate architecture toward the wild-type state, significantly increased bone growth and within 2 weeks of treatment corrected the MCDS-induced hip distortion. These results indicate that CBZ is likely to be effective in ongoing clinical trials against all forms of MCDS whether caused by premature stop codons or substitutions.

## Introduction

Metaphyseal chondrodysplasia type Schmid (MCDS) is a mild, genetically dominant dwarfism caused by mutations in type X collagen (16,17). Most MCDS-causing mutations identified to date are clustered within the C-terminal NC1 trimerization domain of collagen X. These mutations are almost equally divided between missense mutations and mutations that introduce a premature termination signal either directly or as a result of a frameshift (2). Direct analysis of cartilage tissues from two MCDS patients with the non-sense mutations Y632X and W611X revealed complete degradation of the mutant mRNAs most likely through a mechanism similar to but distinct from the classical non-sense-mediated mRNA decay (NMD) (1,3). In an MCDS proband with the Y663X mutation, 64% wild-type and 36% mutant mRNA transcripts were found in a cartilage growth plate biopsy (9). In the unconventional form of NMD that degrades collagen X mRNA in chondrocytes, the 3′ untranslated region (UTR) of the mRNA is used to help recognize mutant mRNA instead of intron–exon boundaries markers usually employed in NMD (14).

Although the importance of RNA surveillance and quality control imposed by the classical NMD process is well established in the molecular pathology of many genetic disorders (4,7), the role and functional significance of the collagen X-specific form of NMD in the pathogenesis of MCDS remains largely unknown, particularly, as increased endoplasmic reticulum (ER) stress appears to play a pivotal role in the disease mechanism in most cases examined to date (9,13,15). To address this issue, we generated a knock-in mouse model expressing the mouse equivalent of *Col10a1* p.Y632X MCDS-causing mutation. Surprisingly, the mutant collagen X mRNA was stable and present at the same level as the wild-type mRNA. Nevertheless, the mouse heterozygous for the mutation exhibited a robust MCDS phenotype characterized by reduced endochondral bone growth rate and a hip dysplasia, marked expansion of the hypertrophic zone (HZ) and intracellular retention of misfolded mutant proteins within the cartilage growth plate hypertrophic chondrocytes (HCs). This was accompanied by an increased ER stress and a marked unfolded protein response (UPR) that disrupted the highly coordinated differentiation process of HCs, similar to previous mouse models for MCDS (13,15). Treatment of this MCDS mouse with carbamazepine (CBZ), a drug recently shown to reduce the disease severity in the *Col10a1* p.N617K mouse line (11), reduced ER stress in the growth plate, restored growth plate architecture toward the wild-type state, significantly boosted bone growth rates and within 2 weeks of treatment corrected the MCDS-induced hip distortion.

## Results

### Generation of collagen X (p.Tyr632stop) MCDS mouse model

**Figure 1:**
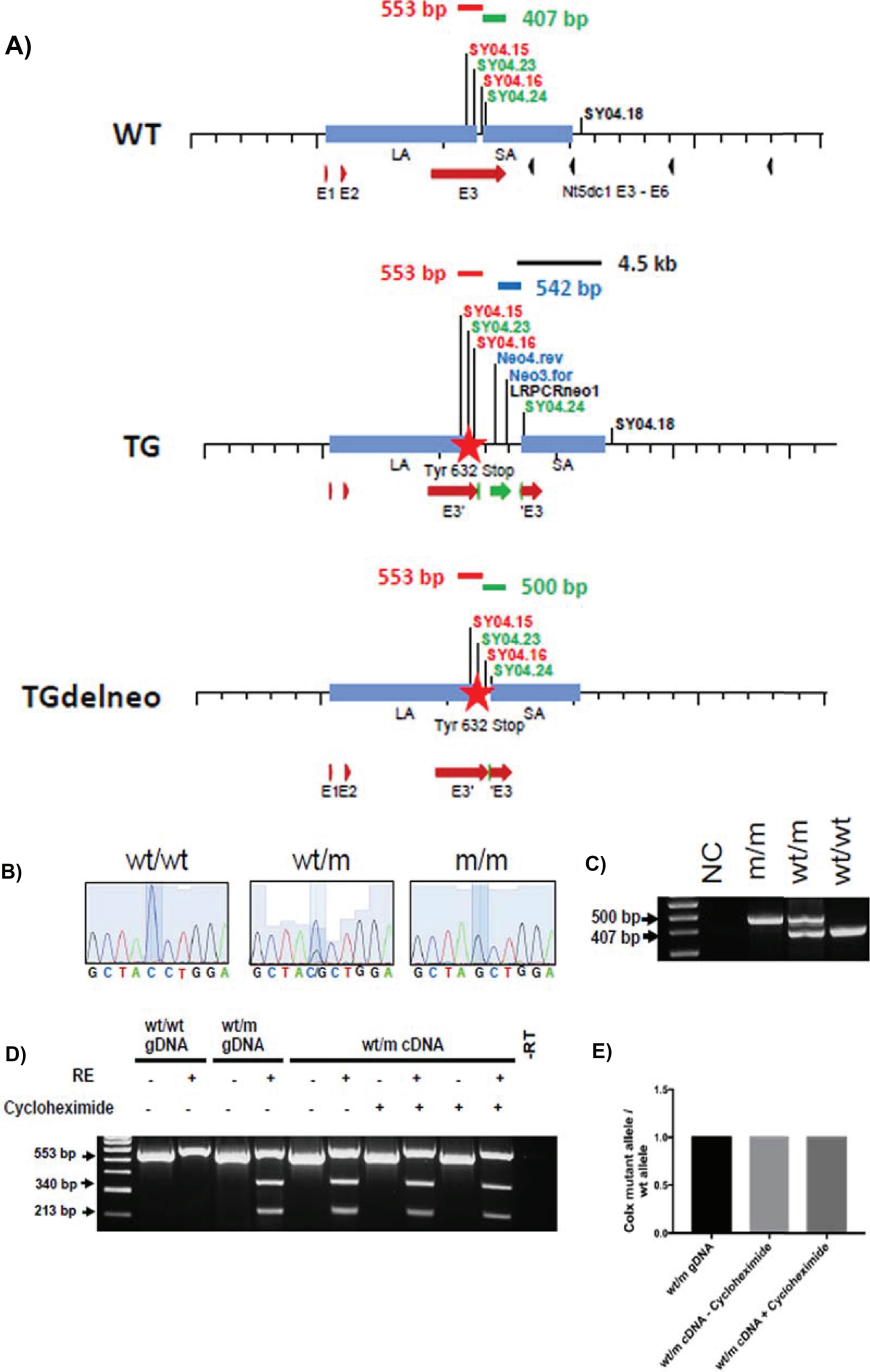
Generation of *Col10a1* p.Tyr632stop MCDS mouse model. (**A**) The different *Col10a1* alleles. Comparison of the *Col10a1* wild type (WT, upper panel), the targeted allele (TG, middle panel) and the targeted allele after deletion of the neomycin cassette (TGdelneo, lower panel). The binding sites of the primers used for genotyping are indicated in relation to the *Col10a1* exons (red arrows), the Nt5c1 exons (black arrow heads—an adjacent gene on the chromosome) and the FRT flanked neomycin cassette (green). The corresponding PCR fragments are indicated in red (for mutation), green (for neo deletion) and blue (for neo presence) and black (for confirmation of homologous recombination). The Tyr632stop mutation inserted in exon 3 of *Col10a1* is indicated as a red star. A short residual FRT insert remains 199 bp 3′ of the wt stop codon in the 3′ UTR of the *Col10a1* gene at the site of the neo gene insertion following its deletion (see Materials and Methods). (**B**) Chromatograph of the sequence flanking the mutation site, showing the C>G single-base pair substitution that changes the Tyr^632^ codon (TAC) to a stop codon (TAG) in the heterozygote (wt/m), represented by a double peak, and the single G peak in the mouse homozygous for the mutation (m/m). (**C**) Genotyping was performed by PCR amplification across the remaining *FRT* site left after *Flp*-mediated deletion of the FRT-flanked neomycin cassette using F and R primers shown in (**A**). (**D**) RT-PCR on the ribs cartilage growth plate sample from 3-week-old heterozygous mice that were cultured in the presence or absence of 100 μg/ml cycloheximide for 4 h, and restriction enzyme digestion (RE) with *Nhe*I restriction enzyme to assess NMD. Genomic DNAs (gDNA) from wild-type and heterozygous mice serves as positive controls. (−RT = minus reverse transcriptase control). The wild-type allele was observed as a 553 bp band, while Y632X mutant allele was cut into two bands of 340 and 213 bp sizes following RE digestion with *Nhe*I. (**E**) Quantification of the ratio of collagen X mutant allele / wild-type allele in cDNA from heterozygous mice before and after incubation with cycloheximide relative to their levels in the heterozygous genomic DNA as described in Materials and Methods.

The mouse model of the human collagen X Tyr632stop mutation was generated by introducing the mutation via embryonic stem cell based targeting. For this, a tissue-culture selection cassette (Flp/FRT excisable neomycin resistance) was co-introduced into the 3′ UTR of exon 3 (Fig. 1A) and subsequently excised following successful targeting. A short residual FRT site remains in the 3′ UTR of the *Col10a1* gene at the site of the neo gene insertion following its deletion.

### Collagen X p.Tyr632stop mouse mRNA did not display NMD

In direct analysis of growth plate cartilage mRNA from an MCDS patient heterozygous for *COL10A1* p.Y632X mutation, expression of only the normal allele was detected (3). The absence of mutant mRNA in this biopsy from an MCDS patient raised the possibility that the pathology may result from type X collagen haploinsufficiency (3). To examine further the disease mechanism associated with this mutation, we looked for unstable mutant *Col10a1* mutant mRNA using the cycloheximide method used previously (1). Following reverse transcription polymerase chain reaction (RT-PCR), the ratio of mutant:wt mRNA was quantified and normalized against their levels in the heterozygous genomic DNA as described in Materials and Methods. No increases in the ratio of mutant:wt mRNA in the cartilage growth plate samples from either ribs (Fig. 1D and E), knee and arm joints (data not shown) of 3-week-old wt/m MCDS mouse model were apparent following cycloheximide incubation. Indeed, the ratio of mutant:wt *Col10a1* mRNA was 1:1 with or without preincubation in cycloheximide (Fig. 1D and E), indicating that the mutant mRNA had the same stability as wt mRNA and, unlike the human equivalent, the mutant mRNA in this mouse model was not subject to a form of NMD. See Discussion for possible reasons for this difference between the mouse model and the human situation.

### Mice heterozygote (wt/m) and homozygote (m/m) for *Col10a1* p.Y632X mutation displayed a robust MCDS phenotype

**Figure 2:**
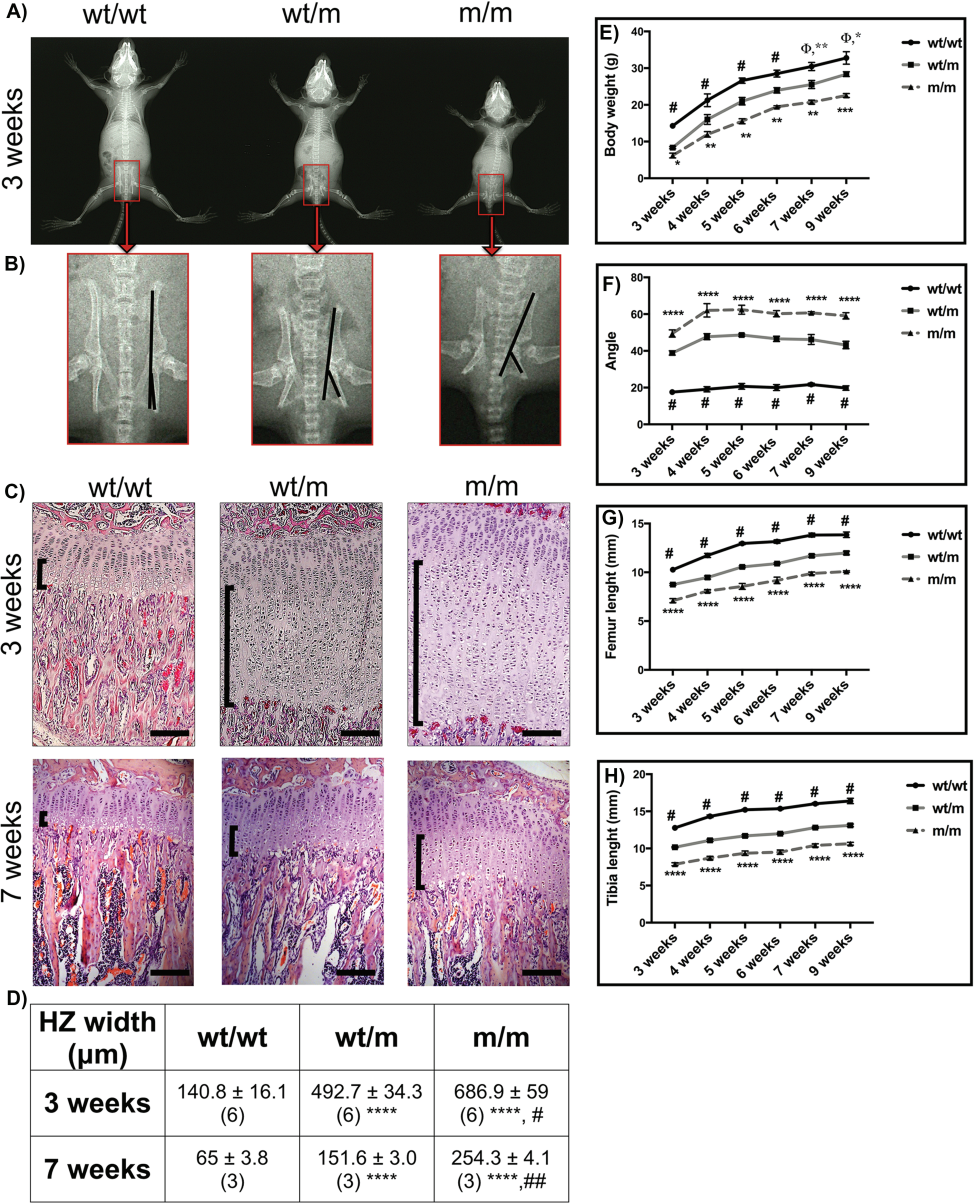
Analysis of the cartilage growth plate and morphometric characterization of *Col10a1* p.Tyr632stop MCDS mouse model. (**A**) X-ray images of female mice at 3 weeks of age. (**B**) The expanded view of the indicated areas from pelvis in (A) showing distortion of the ischial tuberosity in wt/m and m/m mice. (**C**) H&E staining of tibial growth plates at 3 and 7 weeks of age. The HZ is indicated by the vertical back brackets. (**D**) HZ widths at specified time points. ^****^P < 0.0001 compared with wt/wt controls, ^#^*P* < 0.05 and ^##^*P* < 0.0001 compared with wt/m. (**E**–**H**) Comparison of (E) body weight, (F) the angle of deflection of the ischial tuberosity, (G) femur length and (H) tibia length between wt/wt, wt/m and m/m genotypes over a 9-week period. Mean ± SEM (*n* ≥ 5 for each time point). ^*^P < 0.05, ^**^P < 0.01 and ^***^P < 0.001 compared with wt/m; ^****^P < 0.0001 compared with both wt/wt and wt/m; ^#^*P* < 0.0001 compared with wt/m; ^Φ^*P* < 0.0001 compared with m/m. Scale bars are 100 μm.

Both wt/m and m/m mice grew slower than their wt/wt littermates from birth (data not shown). The differences between wt/wt and both wt/m and m/m mice body weights (Fig. 2E) and endochondral bone growth rates (Fig. 2G and H) were maintained over the time course of the experiment and were still apparent at 9 weeks of age. The lengths of femur and tibia bones as well as body weight in wt/m mice were intermediate between the wt/wt and m/m values (Fig. 2E, G and H). The inner canthal distance (ICD) between the eye sockets, a part of the skeleton formed by intramembranous ossification, was identical between all genotypes (data not shown), indicating that the observed skeletal defects in wt/m and m/m mice are specifically related to defects in endochondral bone growth.

Another feature of the MCDS pathology in mouse is the presence of hip dysplasia, which is characterized by an increase in the angle of deflection of the ischial tuberosity (11,13). The angle of deflection was increased approximately 2-fold in wt/m mice compared with their wt/wt control littermates at 3 weeks of age (Fig. 2B and F). There was a further significant 1-fold increase in this angle in 3-week-old m/m mice compared with their age-matched wt/m and wt/wt littermates. This trend was maintained throughout the time course of experiment (Fig. 2B and F).

Expansion of the cartilage growth plate HZ is the histological hallmark of MCDS (13,15). The HZs of tibial growth plates were significantly expanded by 3.5- and 5-fold in 3-week-old wt/m and m/m mice, respectively, compared with their wt/wt control littermates (Fig. 2C and D). Such marked expansions of HZ in both wt/m and m/m mice compared with wt/wt mice were also present at birth (data not shown) and were still apparent at 7 weeks of age despite marked reductions in the overall height of growth plates in all genotypes that occur naturally as the animals mature (Fig. 2C and D).

**Figure 3:**
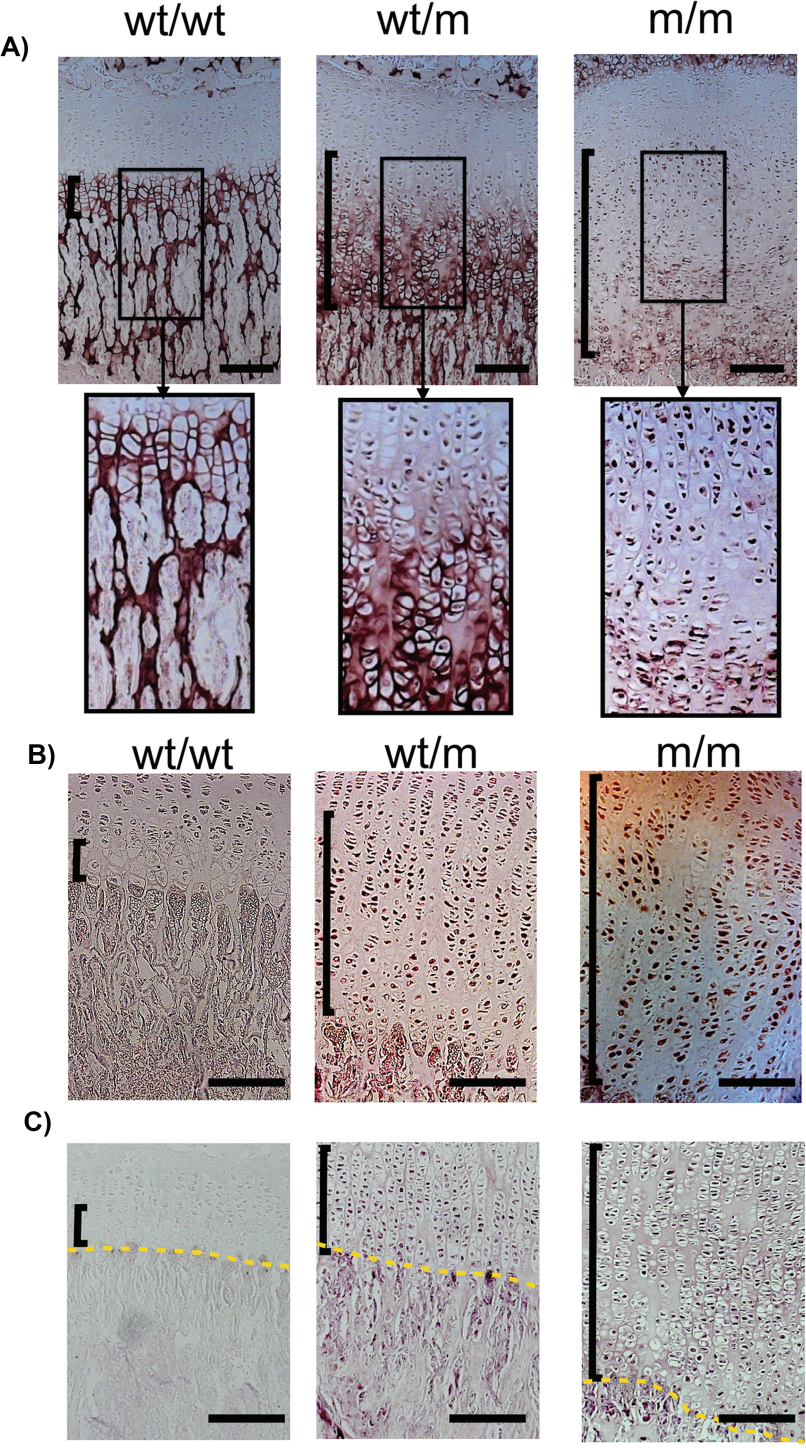
Immunohistological characterization of 3-week-old tibial growth plates. IHC for (**A**) collagen X, (**B**) Bip and (**C**) Creld2. The vertical black brackets delineate the HZs. The yellow dashed line is the vascular invasion front. The black boxed photomicrographs represent an expanded view of the indicated areas within HZs in the sections from specified genotypes. Scale bars are 100 μm.

The MCDS phenotype was shown to be a direct result of increased ER stress caused by intracellular retention of mutated and misfolded collagen X protein within the HCs that disrupts HC differentiation (13,15). Immunohistochemical analysis of tibial growth plates in wt/wt mice revealed the expected secretion of collagen X into the extracellular matrix surrounding the HCs. The mutant protein in m/m mice was mainly retained intracellularly with a significantly delayed and reduced collagen X secretion in the lower half of the HZ (Fig. 3A). wt/m mice displayed an intermediate phenotype with less retention and more secretion particularly in the lower half of the HZ compared with the m/m mouse (Fig. 3A). Immunostaining for the ER stress-induced chaperones GRP78/BiP and Cysteine Rich with EGF Like Domains 2 (Creld2) was markedly induced in the HCs of wt/m and m/m animals compared with the wt/wt control(Fig. 3B and C).

### Increased ER stress and UPR in mice expressing Col10a1 p.Y632X mutation

We assessed the overall level of ER stress by measuring the level of known markers for ER stress such as *Bip*, *Chop* and *Creld2* mRNA and activities of the three canonical signalling pathways of UPR by measuring the levels of Activating transcription factor 4 (Atf4) protein (for protein kinase R (PKR)-like endoplasmic reticulum kinase (PERK) activity), the cleaved form of Atf6α(N) (for Activating transcription factor 6 (ATF6) activity), and the level of *Xbp1* mRNA splicing (for inositol requiring enzyme-1 (IRE1) activity).

**Figure 4:**
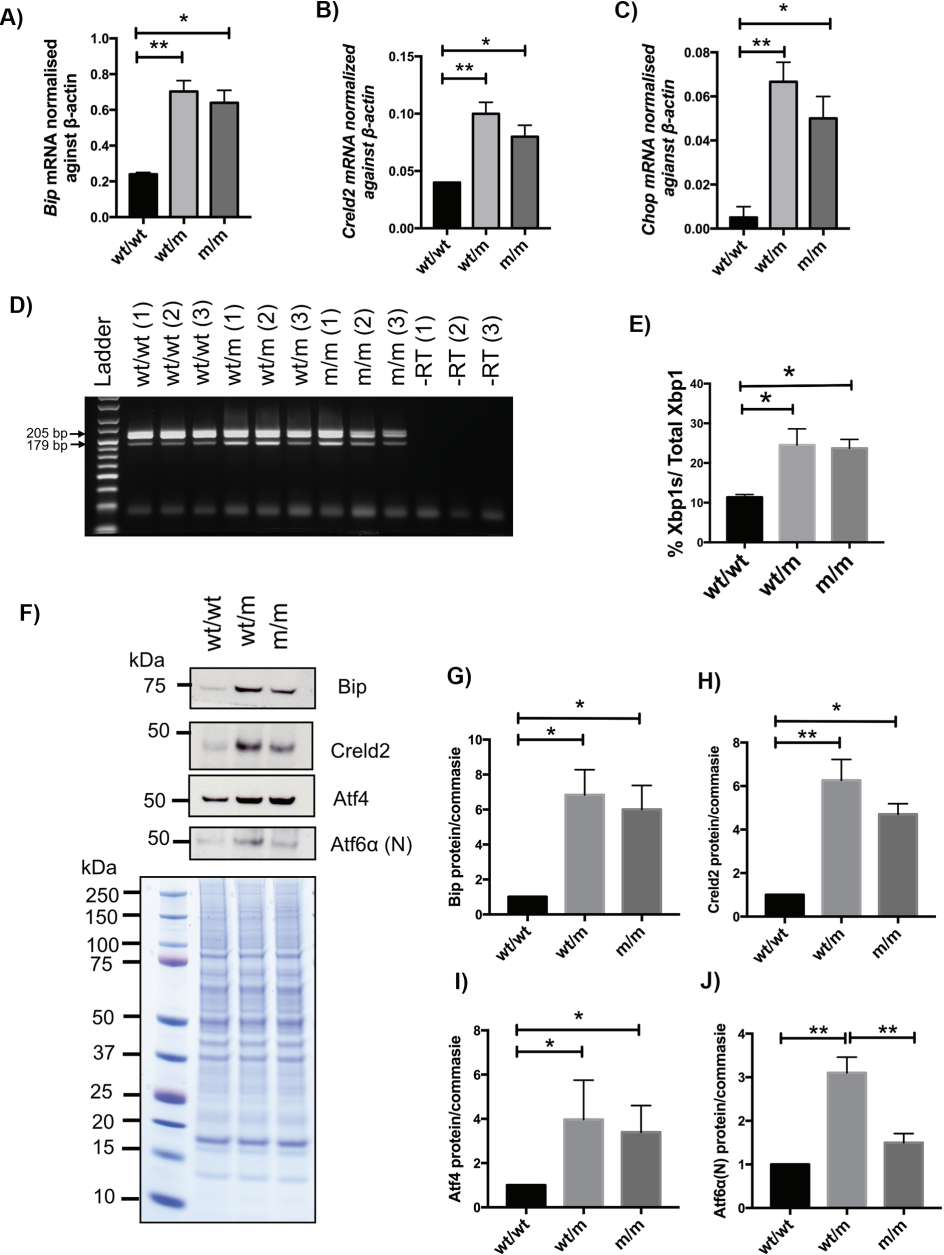
Assessment of ER stress in *Col10a1* p.Tyr632stop MCDS mouse model. The cartilage growth plate extracts from three 21-day-old mice were analysed with qPCR for the expression of (**A**) *Bip*, (**B**) *Creld2* and (**C**) *Chop* mRNAs. (**D**) RT-PCR on cDNA drive form three pooled ribs growth plate extracts of 21-day-old mice to detect the unsliced form of Xbp1 (size = 205 bp FL) or the active and spliced form of Xbp1 (Xbp1s) with the size of 179 bp (−RT = minus reverse transcriptase control). (**E**) The average rate of Xbp1 splicing from three independent samples for each genotype. (**F**) A typical western blots of rib growth plate extracts at 3 weeks of age for Bip, Creld2, Atf4 and cleaved and activated form of Atf6α and Atf6α(N), proteins. Coomassie blue-stained gel was used as loading control. (**G**–**J**) Quantification of (**G**) Bip, (**H**) Creld2, (**I**) Atf4 and (J) Atf6α(N) from three independent experiments. All statistical analyses by ANOVA. (^*^P < 0.05, ^**^P < 0.01).

The expression of *Bip*, *Creld2* and *Chop* mRNAs was significantly increased in rib cartilage growth plate samples from 21-day-old wt/m and m/m mice compared with their wt/wt littermates (Fig. 4A–C). The levels of *Xbp1s* mRNA were increased significantly, approximately 2-fold, in the growth plates of 3-week-old wt/m and m/m mice compared with their wt/wt controls (Fig. 4D and E).

At the protein level, Bip and Creld2 proteins were significantly induced in wt/m and m/m rib growth plate extracts when compared with their wild-type controls at 3 weeks of age (Fig. 4F–H), in agreement with the increased immunostaining noted previously (Fig. 3B and C). In addition, the levels of Atf4 protein and the cleaved form of Atf6α(N) were both significantly increased in the wt/m and m/m growth plate extracts (Fig. 4F, I and J). These data indicate that the *Col10a1* p.Y632X mutant protein induces a significant UPR involving PERK, ATF6 and IRE1 signalling.

### Y632X MCDS-causing mutation disrupted hypertrophic differentiation

**Figure 5:**
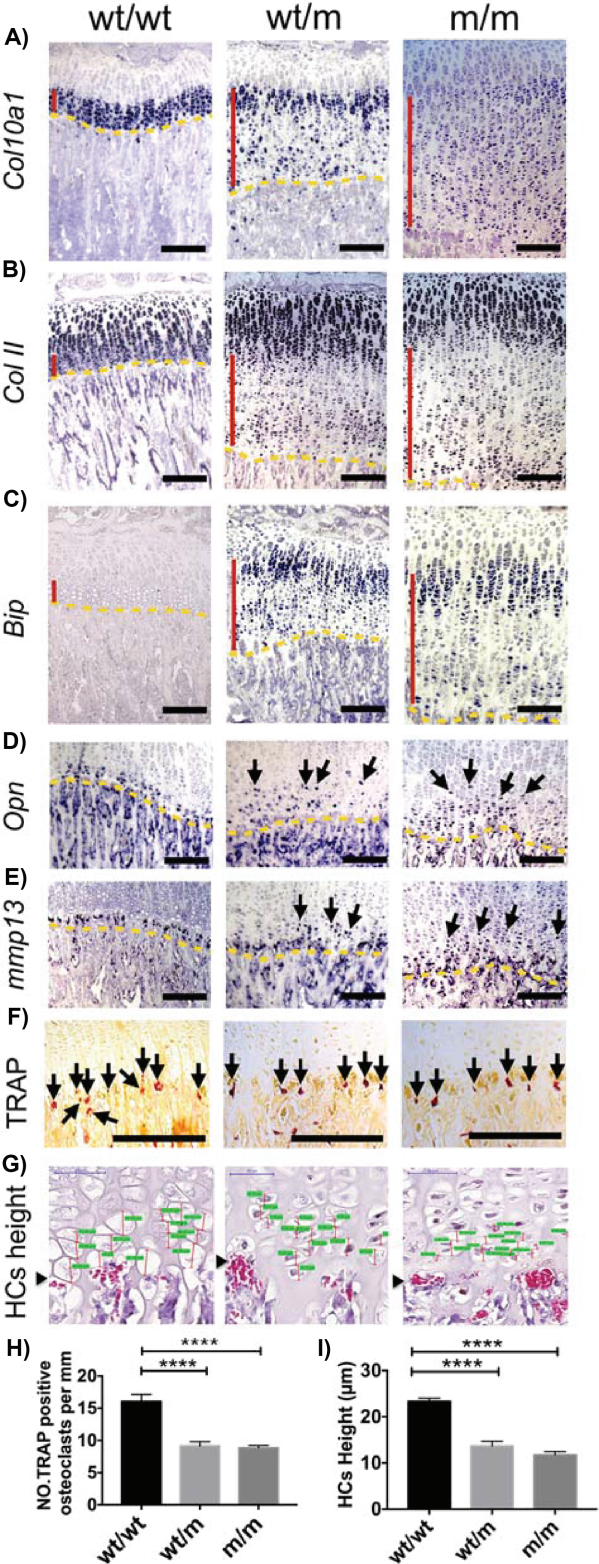
Histological characterization of 3-week-old tibial growth plates. ISH for (**A**) *Col10a1*, (**B**) *Col II*, (**C**) *Bip*, (**D**) *Osteopontin* (arrows) and (**E**) *Mmp13* (arrows). The presence of transcript is indicated by the dark blue staining. The HZ is indicated by the vertical red line and the vascular invasion front by the yellow dashes. (**F**) TRAP staining for osteoclasts at the vascular invasion front (arrows). (**G**) Snapshot of measurement of height of the most terminal hypertrophy chondrocytes (HCs). HC heights are indicated by the red vertical lines and their corresponding measurements are highlighted in green. (arrowhead = vif). (**H**) Number of osteoclasts per mm of vascular invasion front. Mean ± SEM (4). (**I**) The height attained by HCs adjacent to vascular invasion front. Mean ± SEM (5) (^****^P < 0.0001 as determined by one-way ANOVA). Scale bars are 100 μm.

As has been noted in other MCDS models, increased ER stress in HCs of the Y632X mouse (wt/m and m/m) caused a disruption in the pattern of collagen X expression with a sporadic on–off distribution in cells in the lower parts of the HZ (Fig. 5A); an inappropriate sporadic re-expression of collagen II mRNA in the lower parts of the HZ (Fig. 5B); a marked induction of *BiP* mRNA in the top part of the HZ with sporadic expression lower down (Fig. 5C); and sporadic uncoordinated expression of both *Opn* and *Mmp13* mRNAs by cells in the lower part of the HZ, whereas expression of these two mRNAs is limited to the most terminally differentiated cells immediately adjacent to the vascular invasion front in the wt/wt controls (Fig. 5D and E). In addition, recruitment of osteoclasts [tartrate-resistant acid phosphatase (TRAP)-positive cells] to the vascular invasion front and the height achieved by the terminal HCs were both significantly reduced in wt/m and m/m cells (Fig. 5F–I). All these data indicate a disrupted hypertrophic differentiation in the growth plates of mice expressing the truncated mutant protein.

### CBZ significantly reduced disease severity in the Col10a1 p.Y632X mouse

**Figure 6:**
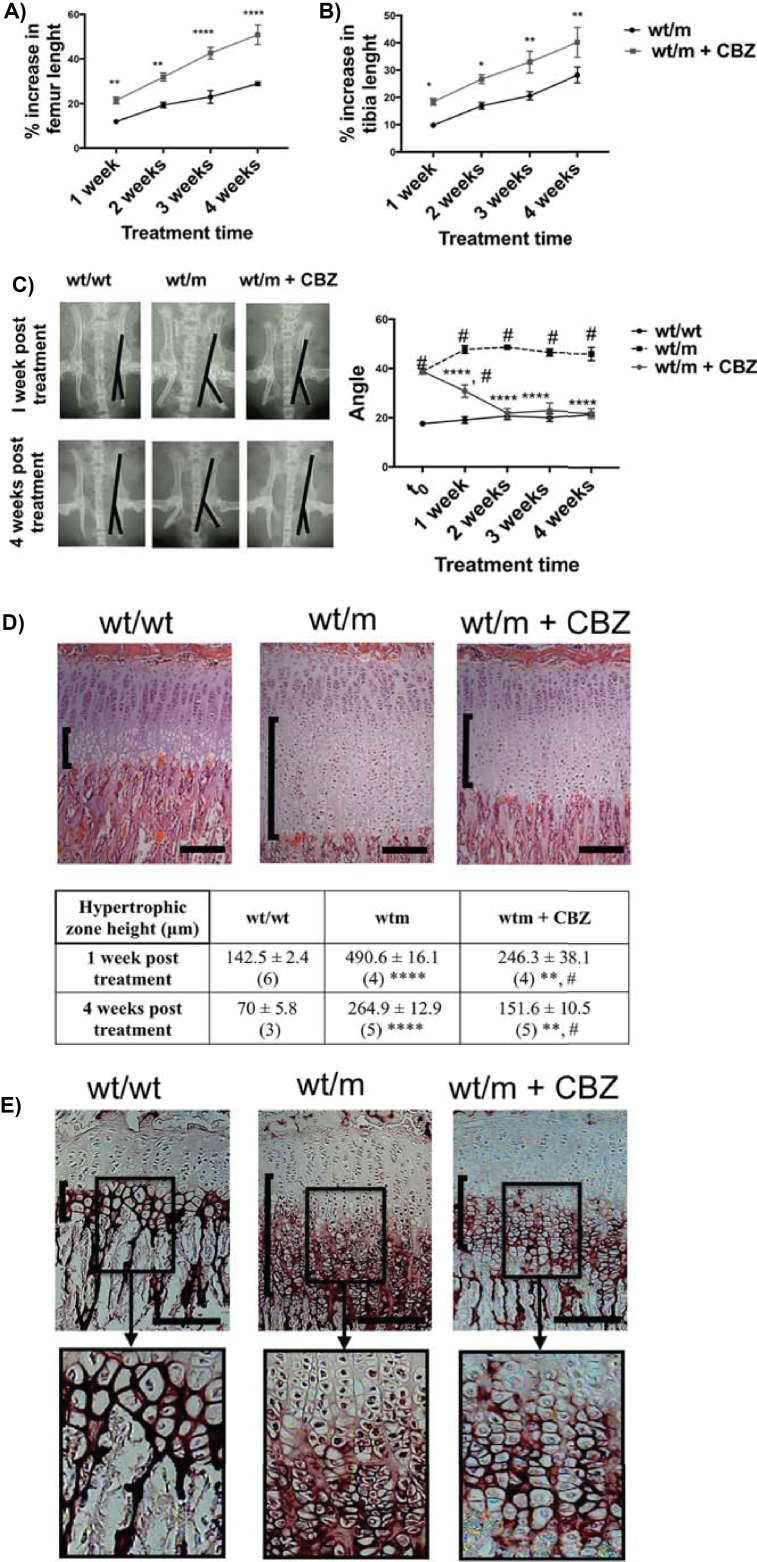
Effect of carbamazepine on the MCDS-associated characteristics. Three-week-old wt/m mice were treated with CBZ for a period of 1–4 weeks. Untreated wt/m mice and mice wild type for collagen X (wt/wt) were used as controls. Percentage of increase in the length of (**A**) femur and (**B**) tibia bones based on length at 3 weeks of age in each animal. Mean ± SEM (*N* ≥ 4) at each time point (^*^P < 0.05, ^**^P < 0.01, ^****^P < 0.0001 as determined by one-way ANOVA). (**C**) X-ray images of pelvis from 4-week-old mice with specified genotypes illustrating the angle of deflection of the ischial tuberosity in wt/m and wt/m + CBZ mice. This angle was measured for each group (mean ± SEM, *N* ≥ 5 at each time point (^#^*P* < 0.0001 compared with wt/wt, ^****^P < 0.0001 compared with wt/m). (**D**) H&E staining of tibial growth plates at 4 weeks of age. The HZ is indicated by the vertical back brackets. Mean ± SEM (*N*). ^**^P < 0.01 and ^****^P < 0.0001 compared with wt/wt; ^#****^P < 0.0001 compared with wt/m as determined by one-way ANOVA. (**E**) IHC for collagen X. The vertical black brackets delineate the HZs. The black boxed photomicrographs represent an expanded view of the indicated areas within HZs in the sections from specified genotypes. Scale bars are 100 μm.

wt/m mice were treated with CBZ (250 mg/kg bw/day) by oral gavage for 1–4 weeks. This dose level in mice is equivalent to that used in treating epilepsy and related disorders in humans taking into account species differences (12). One week of CBZ treatment significantly increased the femur and tibia growth in wt/m MCDS mice (Fig. 6A and B), and this effect continued throughout the 4-week treatment period. While 1 week of CBZ treatment was sufficient to significantly reduce the increased ischial tuberosity angle of deflection in wt/m mice, 2 weeks of treatment restored the hip geometry to that seen in the wt/wt control mice (Fig. 6C). In addition, both 1 and 4 weeks of CBZ treatment reduced the MCDS-associated expanded HZ width in wt/m mice by half (Fig. 6D).

Treatment with CBZ had a quiet dramatic effect on the localization of the collagen X protein within the cartilage growth plate in mice expressing Y632X MCDS-causing mutation. One week of CBZ treatment reduced the intracellular retention of mutant collagen X and improved the organization of the hypertrophic cell columns and their surrounding collagen X matrix (Fig. 6E).

### CBZ treatment reduced ER stress in the *Col10a1* p.Y632X mouse growth plate

**Figure 7:**
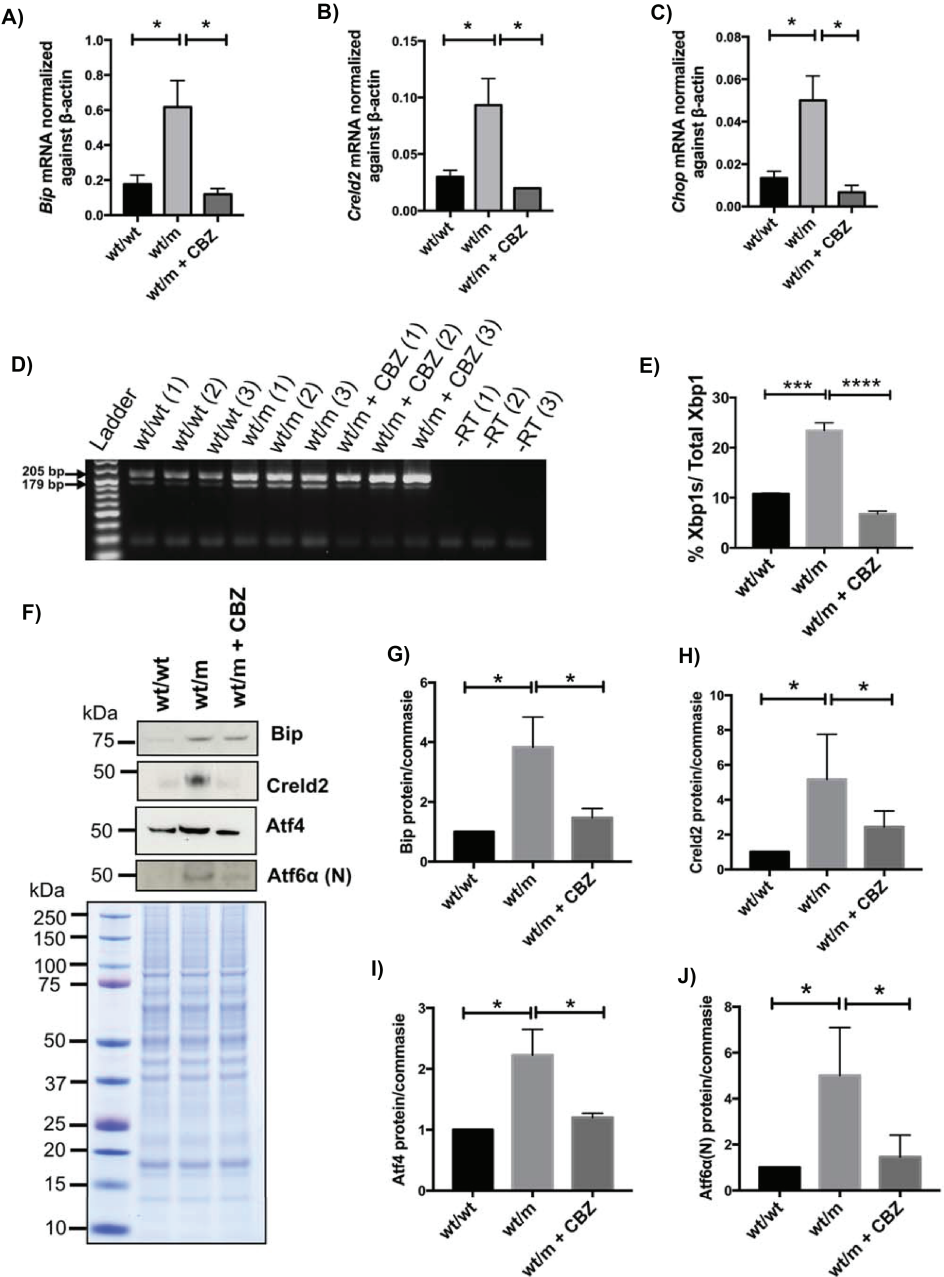
Effect of carbamazepine on the MCDS-associated increased ER stress. Three 3 -week-old wt/m mice were treated with CBZ for 1 week. The cartilage growth plate extracts from these mice were analysed with qPCR for the expression of (**A**) *Bip*, (**B**) *Creld2* and (**C**) *Chop* mRNAs. (**D**) RT-PCR on cDNA derived from **t**hree pooled rib growth plate extracts of 1-week CBZ-treated mice and controls to detect the unsliced form of Xbp1 (size = 205 bp) or the active and spliced form of Xbp1 (Xbp1s—179 bp). −RT = minus reverse transcriptase control). (**E**) The average rate of Xbp1 splicing from three independent samples for each genotype. (**F**) A typical western blot of rib growth plate extracts at 4 weeks of age for Bip, Creld2, Atf4 and cleaved and activated form of Atf6α and Atf6α(N), proteins. Coomassie blue-stained gel was used as loading control. (**G**–**J**) Quantification of (**G**) Bip, (**H**) Creld2, (**I**) Atf4 and (**J**) Atf6α(N) from three independent extracts. All statistical analyses by ANOVA (^*^P < 0.05, ^****^P < 0.0001). Scale bars are 100 μm.

One week of CBZ treatment significantly reduced induction of known ER stress markers *Bip*, *Creld2* and *Chop* mRNAs in the rib growth plate extracts of 4-week-old wt/m mice (Fig. 7A–C). Likewise, the splicing of *Xbp1* mRNA was also suppressed by 1 week of CBZ treatment (Fig. 7D and E). In addition, CBZ treatment significantly reduced the elevated levels of Bip and Creld2 proteins in rib growth plate extracts assessed by western blotting (Fig. 7F–I). Furthermore, treatment with CBZ reduced the induction of Atf4 (indicative of PERK signalling) (Fig. 7F and I) and of cleaved Atf6α (Fig. 7F and J). These data clearly demonstrate that a short period of treatment with CBZ was sufficient to reduce the elevated ER stress and enhanced activities of UPR signalling pathways in wt/m mice.

### CBZ treatment improves hypertrophic differentiation in the growth plate of the *Col10a1* p.Y632X mouse

**Figure 8:**
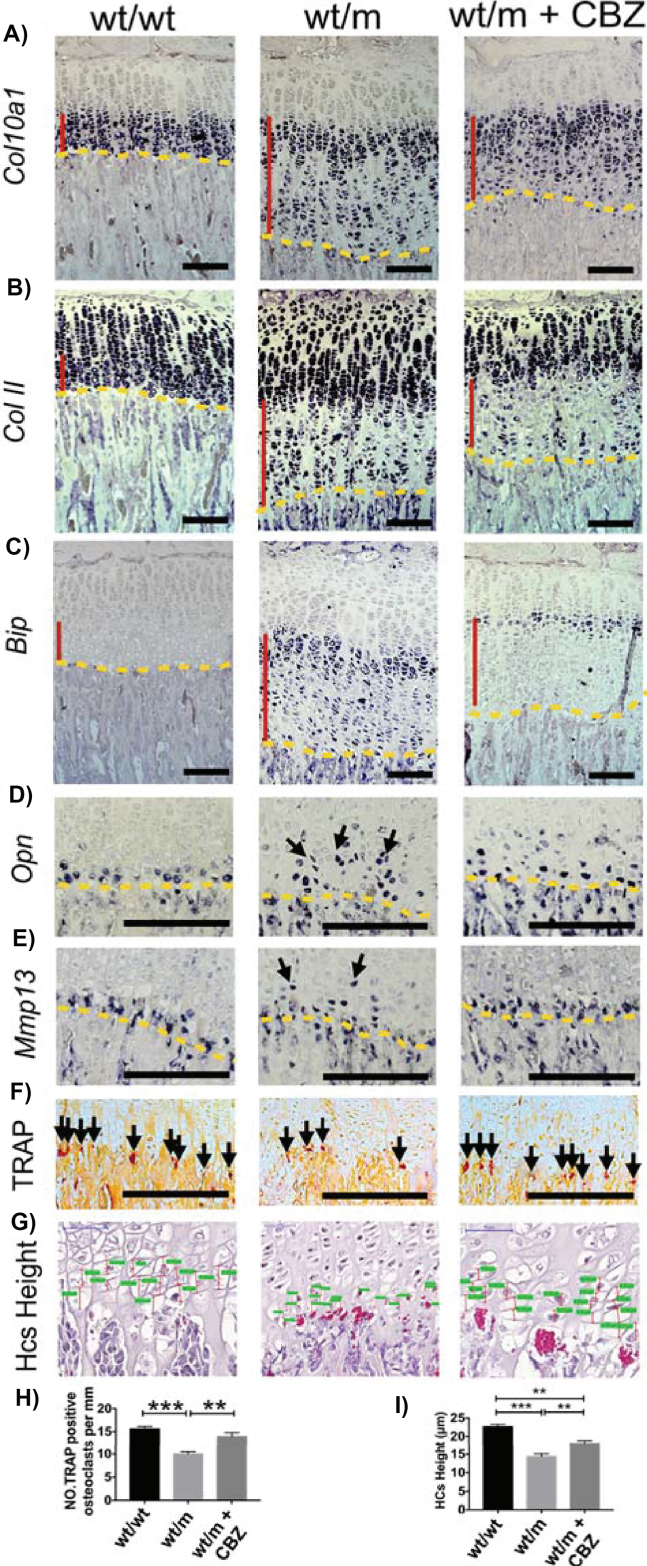
Effect of carbamazepine on the MCDS-associated altered differentiation of chondrocytes. Three-week-old wt/m mice were treated with CBZ for 1 week. ISH for (**A**) *Col10a1*, (**B**) *Col II*, (**C**) *Bip*, (**D**) *Osteopontin* (arrows) and (**E**) *Mmp13* (arrows). The presence of transcript is indicated by the dark blue staining. The HZ is indicated by the vertical red line, and the vascular invasion front by the yellow dashes. (**F**) TRAP staining for osteoclasts at the vascular invasion front (arrows). (**G**) The final height attained by hypertrophic chondrocytes adjacent to vascular invasion front. (**H**) Number of osteoclasts per mm of vascular invasion front. Mean ± SEM (*N* = 4). (**I**) Heights of the most terminal HCS**.** Mean ± SEM (5) (^**^P < 0.01, ^***^P < 0.001 as determined by one-way ANOVA). Scale bars are 100 μm.

CBZ treatment of wt/m mice reduced the disrupted expression patterns of collagen X and collagen II mRNAs in the HZ (Fig. 8A and B). *Bip* mRNA induction was markedly reduced in the CBZ-treated MCDS mouse growth plate and limited in large part to the early HCs (Fig. 8C). CBZ treatment of wt/m mice partially corrected the disrupted *Opn* mRNA expression (Fig. 8D) and normalized the expression of *Mmp13* mRNA (Fig. 8E). CBZ treatment of wt/m mice significantly enhanced the osteoclast requirement to the vascular invasion front (Fig. 8F and H) and also markedly improved chondrocyte hypertrophy based on the height of the terminal HCs (Fig. 8G and I). These data clearly indicate that CBZ treatment significantly corrected the aberrant differentiation of HCs apparent in the wt/m mice.

## Discussion

There are several unusual aspects of message instability of mutant *Col10a1* mRNA containing premature stop codons that distinguish the process seen in MCDS from classical NMD including tissue specificity, the presence of the stop codon in the last exon and involvement of the 3′ UTR region in the instability mechanism (3,5,14). The fact that in two separate individuals with MCDS this mechanism appeared capable of destroying all the mutant mRNA raises the question whether the disease mechanism in these cases is different from the ER stress-based mechanism described previously that is triggered by the presence of the mutant protein. This is an important question, as we have recently described a potential treatment for MCDS based on reducing ER stress (11) that may not be effective for those cases of MCDS caused by alleles with mRNA instability if the disease mechanism associated with the latter is significantly different. A previously reported transgenic mouse model expressed a *Col10a1* p.P620fsX621 MCDS allele that was subject to some degree of instability, but the remaining mutant mRNA from the transgene was translated and the mutant protein was produced and, hence, ER stress was detected in the collagen X-expressing cells (9). Only if you have complete destruction of the mutant mRNA and therefore only a small amount of mutant protein associated with the pioneer round of translation required for mRNA destabilization would it be possible to determine whether the mechanism generating the MCDS pathology in these cases featuring mRNA instability differs from the ER stress mechanism associated with the expression of mutant collagen X protein.

We therefore generated a gene-targeted mouse model expressing the mouse equivalent of one of the mutations associated with complete mRNA instability in humans, namely the *COL10A1* p.Tyr632stop mutation (3). However, in this mouse model, the mutant mRNA was as stable as the wild-type mRNA (Fig. 1D and E) and there was no evidence of mRNA instability. The mRNA mechanism is active in the mouse, as some, but not complete, instability of mRNA was found in the transgenic mouse described earlier (9). One possible explanation for the lack of instability of the *Col10a1* p.Y632X mRNA in the current mouse model is the presence of a short inserted sequence including a residual *FRT* site in the 3′ UTR of the mutant mRNA left after deletion of the targeting selection cassette. The structure of parts of the 3′ UTR region has been shown to be important for the destabilization of collagen X mRNA containing premature stop codons (5,14). Although the selection cassette for gene targeting was deliberately placed in a non-conserved sequence, it is still possible that the presence of the short residual sequence insert, left after *Flp* recombinase-mediated deletion of the cassette, disrupted the 3′ UTR-based recognition system for destabilization.

Although C*ol10a1* p.Y632X mice did not show mRNA instability, they displayed typical characteristics of MCDS such as short limb dwarfism, hip dysplasia and expansion of HZ (Fig. 2). The mutant mRNA was expressed and retained intracellularly within the HCs, eliciting a marked increase in ER stress and a strong UPR (Figs 3 and 4). In addition, the Y632X mouse growth plate exhibited the same pattern of disruption to HC differentiation associated with previous MCDS mouse models (6,9,13,15). The current study therefore demonstrates that when translated into protein, the Y632X mutant collagen X mRNA misfolds and produces an MCDS phenotype by the same mechanism as has been previously reported.

Previous studies have clearly demonstrated a gene dosage effect in which the level of mutant collagen X expression positively correlates with disease severity (9,13,15). Likewise, in this study, wt/m mice had significantly milder phenotype than their homozygous counterparts. However, it is noteworthy that the MCDS phenotype in the Y632X mouse model was significantly more marked than our previous N617K mouse (11,13). The wt/m N617K mouse had a mild phenotype, and the m/m equivalent had a more severe MCDS phenotype. Indeed, in our recent report on the effects of CBZ, an ER stress-reducing agent, upon the MCDS phenotype, we treated mice homozygous for *Col10a1* p.N617K mutation because of their more marked phenotype. The phenotype of the mouse heterozygous for the Y632X mutation reported in this article is significantly more severe than that of the mouse homozygous for the previously reported N617K collagen X mutation.

In the current study, we therefore treated mice heterozygous for the Y632X mutation with CBZ, as this most closely mirrors the treatment in humans where individuals diagnosed with MCDS are almost always heterozygous for the mutant *COL10A1* allele. CBZ treatment for 1 week dramatically resolved intracellular retention of misfolded mutant collagen X proteins within the HCs (Fig. 6E), significantly attenuated the increased ER stress (Fig. 7) and improved the disrupted differentiation of HCs (Fig. 8). In this regard, some parameters, such as the levels of mRNA coding for the ER stress-related factors such as *Bip*, *Creld2*, *Chop* and *Xbp1s*, were restored to wild-type levels by CBZ treatment, whereas the protein levels for Bip, Creld2, Atf4 and Atf6 remained slightly elevated (Fig. 7). Likewise, osteoclast number at the vascular invasion front and the height of terminal HCs were significantly improved in MCDS mice treated with CBZ (Fig. 8), but the HZ expansion was not completely corrected, showing that the treatment helps alleviate but does not eradicate totally the pathological changes. Four-week treatment with CBZ produced significant increases in endochondral bone growth (Fig. 6A and B). It is particularly noteworthy that within 2 weeks of CBZ treatment commencing, the hip dysplasia apparent in MCDS mice was completely resolved (Fig. 6C).

This is the second MCDS mouse model in which we have demonstrated the potential therapeutic value of CBZ for the treatment of MCDS. Importantly, we demonstrate that the *Col10a1* p.Y632X allele, when translated into protein, generates a severe MCDS phenotype by inducing significant increases in ER stress. Furthermore, the alleviation of ER stress by CBZ treatment significantly reduces the disease phenotype.

It seems likely, as has been suggested previously, that the mRNA instability mechanism displayed by chondrocytes toward collagen X mRNAs encoding C-terminal domain premature termination codons (PTCs) may not be 100% efficient at all stages of development and growth (14). Mutant protein translated from message that has escaped the surveillance mechanism may well be the pathogenic driver of the phenotype, as in each case where such mutant mRNA has been studied *in vivo*, some mutant protein is produced that results in increased ER stress that we now know can be significantly reduced by CBZ treatment. Accordingly, we anticipate that CBZ will be equally effective in the ongoing clinical trial against MCDS caused by either premature stop codons or amino acid substitutions (https://mcds-therapy.eu/).

## Materials and Methods

### Generation of Col10a1 p.Y632X mutant mice

The Tyr632stop mouse mutant allele was generated by ES-cell based targeting. For this, homologous sequences were subcloned from an RPCI23-derived bacterial artificial chromosome RP23-429M5 and the stop mutation was generated by co-introducing an *Nhe*I site. A neomycin selection cassette flanked by FRT sites was inserted 199 bp downstream of the native stop codon into a non-conserved region of 3′ UTR in exon 3 which was devoid of potential microRNA-binding sites. The targeting was performed in C57Bl/6N-derived embryonic stem cells (PRX; Primogenix Inc., St Louis, MO) and confirmed via long-range PCR and sequencing. The FRT-neo-FRT cassette was excised *in vivo* using an Flp recombinase-expressing mouse strain.

### Treatment with CBZ and skeletal analysis

Mice heterozygous for the MCDS-causing Y632X collagen X mutation were X-rayed at 3 weeks of age and orally gavaged with CBZ (250 mg/kg/day) for 1–4 weeks. Mice were anaesthetized using isofluorane and radiographed using a Flaxitron X-ray specimen radiography system (Flaxitron MX-20) and X-ray hyperfilm (GZ28906850, GE Healthcare, Bucks, HP99NA, UK), prior to CBZ treatment and then once per week for a maximum of 4 weeks post CBZ treatment. Mice (wt/wt, wt/m and m/m) were also X-rayed as described earlier at 3 weeks of age and then once per week up to 9 weeks of age. Mice were sacrificed either by cervical dislocation or by carbon dioxide overdose under the provisions of the Animals (Scientific Procedures) Act 1986 and tissues collected for histological analysis. All procedures were carried out according to Home Office regulations.

The lengths of femur and tibia bones and ICDs were measured from scanned radiographic images using the GrowBase software (Certus Technology Associated Limited, Exeter, UK). The bone growth rate was calculated as a percentage increase relative to measurements at 3 weeks of age. The angle of deflection from the vertical tuberosity of the ischium was determined using ImageJ to investigate the severity of the hip dysplasia phenotype. All measurements were analysed by analysis of variance (ANOVA) for statistical significance using GraphPad Prism 6.0 software.

### Histology

The hind lambs were dissected and fixed overnight either in an ice-cold, RNase-free 4% (w/v) paraformaldehyde in diethylpyrocarbonate treated 1× phosphate-buffered saline (PBS) or in 95% ethanol/5% acetic acid (v/v). Bone samples were decalcified in 20% ethyldiaminetetraacetic acid pH 7.4 for 1–14 days depending on the age of the animal. The samples were embedded in paraffin wax and sectioned sagittally using a cool-cut HM 355S microtome (Microm, Micon/VWR, Leicestershire, LE17 4XN, UK), generating 5 μm thick sections. Sections were collected on positively charged superfrost slides (VWR, Leicestershire, LE17 4XN, UK) and dried overnight prior to histological staining, immunohistochemistry (IHC) or *in situ* hybridization (ISH).

For histological, immunohistochemical and *in situ* analyses, at least three animals per genotype were assessed.

### Hematoxylin and eosin staining

Hematoxylin and eosin (H&E) staining was performed as described previously (13), and all images taken using a Carl Zeiss, Cambridge CB13JS, UK AxioVision microscope fitted with an Axiocam colour charge-coupled device (CCD) camera and the associated AxioVision software.

Growth plate zone widths were measured on images of known magnification as described previously (13). The height of PZ was defined from the point where round resting chondrocytes align into columns and become disc shaped to the point where HZ starts. The start of HZ was defined as the time that proliferative chondrocytes stop proliferation, round up and become larger. The vascular invasion front was defined as the end point of HZ. For each animal, five slides at least 75 μm apart were measured and averaged. Measurements were analysed for statistical significance by ANOVA using GraphPad Prism 6.0 software. To measure heights of the most terminal HCs, which are the heights parallel to the direction of growth, four H&E-stained slides per animal, at least 75 μm apart, were scanned using a digital slide scanner (Pannoramic 250 Flash, 3DHISTECH, Sysmex, Bucks, MK88DF, UK) and then analysed by associated panoramic viewer software. For each tissue section, heights of 100 HCs located at the vicinity of vascular invasion front (the last three rows) were measured and averaged. The average height of the most terminal HCs for each animal was defined as the overall average between all related slides. All data were analysed by one-way ANOVA for statistical significance.

### IHC

IHC was performed on 95% ethanol/5% acetic acid fixed sections unless otherwise stated. Sections from paraffin-embedded joints were first deparaffinized in xylene and rehydrated in graded ethanol and water. Antigen unmasking for BiP was carried in citrate buffer pH 6.0 heated to >85°C for 10 min followed by washes in PBS. Antigen unmasking for collagen X IHC was by incubating with 1 mg trypsin (Sigma,~Merck House, Poole, BH15 1TW, UK)/ml of PBS for 12 min at room temperature followed by washes in PBS. Antigen unmasking for CRELD2 IHC was by incubation with 0.2% bovine testicular hyaluronidase (Sigma) in PBS for 15 min at 37°C. Endogenous peroxidase activity was then quenched by incubating with 3% (v/v) hydrogen peroxide in PBS for 5 min. Tissue sections were blocked in PBS containing 2% (v/v) serum derived from the same species in which the secondary antibody was produced and then incubated with primary antibody, each for 1 h at room temperature. Primary antibodies used were collagen X (polyclonal rabbit anti-collagen X against recombinant mouse NC1 domain) diluted 1/500 (1), anti-Grp78 goat polyclonal (SC-1051, Santa Cruz, Insight Biotechnology, Wembley, HA97XX, UK) diluted 1/300, CRELD2 (H3884, R&D Systems, Abingdon, OX143NB, UK) diluted 1/400. Secondary antibodies used were biotinylated goat anti-rabbit IgG (E0432, DakoCytomation Ltd, Aligent, Craven Arms, SY7 8NR, UK) diluted 1/1000 and biotinylated horse anti-goat IgG (BA 9500, Vector Laboratories, Peterborough, PE26XS, UK) diluted 1/1000. Sections were then incubated with ABC reagent (PK-6100, Vector Laboratories, Peterborough, PE26XS, UK) for 30 min and developed using the Vector VIP kit (SK-4600, Vector Laboratories, Peterborough, PE26XS, UK). Slides were dehydrated in increasing concentrations of ethanol and then cleared in xylene and mounted using a xylene-based mounting solution. Images were taken using the Carl Zeiss AxioVision microscope fitted with an Axiocam colour CCD camera and the associated AxioVision software.

### TRAP staining

Osteoclasts at the vascular invasion front were stained using a TRAP staining kit (387A, Sigma-Aldrich) according to the manufacturer’s instructions. The number of positively stained cells (red/brown), indicative of osteoclasts, per millimetre of vascular invasion front was quantified in three sections per animal spaced 50 μm apart. At least three animals per genotype were assessed, and data were analysed by one-way ANOVA for statistical significance.

### ISH

Digoxigenin (DIG)-labelled colourimetric ISH was performed as previously described (13). The resulting complementary DNA (cDNA) probes were cloned into pT7T3, linearized and transcribed using the appropriate restriction enzyme and RNA polymerase.

### Rib growth plate extraction for use in western blotting

Protein extracts from rib growth plates were obtained as described previously (10,13). Briefly, rib cages from 3-week-old mice were dissected and placed in collagenase medium for 1–2 h; growth plates were then dissected form the ribs and cleaned from any remaining muscle under the dissecting microscope. Dissected growth plates from at least three mice were pooled together and placed either in 100 μl 2× sodium dodecyl sulfate polyacrylamide gel electrophoresis (SDS-PAGE) sample buffer for western blotting or in 500 μl of TRIzol (15596018, Life Technologies, Thermo Fisher Scientific, Hemel Hempstead, HP27GE, UK) for RNA extraction. Dissected growth plates were then homogenized using a microdismembranator. For western blotting, the homogenized rib growth plate extracts were boiled in SDS loading buffer containing β-mercaptoethanol and centrifuged. The supernatant was collected, and the total protein concentration was assayed using the Pierce bicinchoninic acid protein assay (# 23227, Thermo Scientific, Hemel Hempstead, HP27GE, UK) with a bovine serum albumin standard curve according to the manufacturer’s protocol. Protein extracts were then analysed by SDS-PAGE and western blotting.

### SDS-PAGE and western blotting

Twenty micrograms of protein was loaded into the precast NuPAGE® Novex® 4–12% Bis-Tris Gels (NP0322BOX, Life Technologies). The gel was electroblotted onto a nitrocellulose membrane, which was blocked 1 h at room temperature with 5% skimmed milk powder in PBS containing 0.1% Tween-20 and 2% (v/v) serum derived from the same species in which the secondary antibody was produced. The membranes were incubated in 1/500 dilution of Grp78/BiP (SC-1051, Santa Cruz), CRELD2 (sc-86110, Santa Cruz) and 1/1000 dilution of ATF6α (37-1) (BAM-73-505-EX) and anti-ATF4 (11815, Cell Signalling, New England Biolabs, Hitchin, SG40TY, UK) primary antibodies in blocking solution overnight at 4°C. Membranes were then incubated with an appropriate horseradish peroxidase-conjugated secondary antibody. An ECL detection kit (Life Technologies) and ECL hyperfilm (GE Healthcare) was used to develop the blots according to the manufacturer’s protocol.

The blots were quantified by densitometry analysis using the ImageJ software on images from scanned films within the linear range of exposure. The intensity of each band was calculated relative to a loading control and standardized against a control protein sample on each blot. The result were analysed by ANOVA for statistical significance using GraphPad Prism 6.0 software.

### PCR analysis to detect Xbp1 splicing

Detection of X-box-binding protein 1 (XBP1) splicing on the rib growth plates from 3-week-old mice were carried out as described previously (8,10). Total RNA from three pooled rib growth plate extracts of 21-day-old mice was extracted (as described earlier). One microgram of purified RNA samples was reverse transcribed to cDNA using the Taqman Reverse Transcription Reagent Kit (Life Technologies) according to the manufacturer’s protocol and subjected to PCR with primers flanking the Xbp1 ER stress-responsive splice site F: 5′-GAACCAGGAGTTAAGAACACG-3′ and R: 5′-AGGCAACAGTGTCAGAGTCC-3′. PCR products, XBP1U with the size of 205 bp and XBP1s with the size of 179 bp, were then separated on 3% agarose gel. The level of XBP1 splicing was then calculated as a percentage of the total XBP1 (XBP1s + XBP1U) using the ImageJ software. To check for any possible contamination with genomic DNA, a minus reverse transcriptase (−RT) control contain all the RT-PCR reagents except the reverse transcriptase was set for each sample.

### Real-time quantitative PCR analysis

Purification of RNA from rib growth plate extracts in TRIzol reagent (Life Technologies) was performed using phenol and chloroform methodology. cDNA was synthesized from 1 μg of purified and DNase-treated (AM1906, Ambion, Thermo Fisher Scientific, Hemel Hempstead, HP27GE, UK) RNA samples using the Taqman Reverse Transcription Reagent Kit (N8080234, Applied Biosystems, Thermo Fisher Scientific, Hemel Hempstead, HP27GE, UK) according to the manufacturer’s protocol. The following primers were used for real-time quantitative PCR (qPCR):

CRELD2 forward 5′-GGGCTGGTGGACAAGTTTAAC-3′ and reverse 5′-CGAATCTCGCTGGACTCGTA-3′, BiP forward 5′-GCTAATGCTTATGGCCTGGA-3′ and reverse 5′-CGCTGGTCAAAGTCTTCTCC-3′, CHOP forward 5′-CCACCACACCTGAAAGCAGAA-3′ and reverse 5′-AGGTGCCCCCAATTTCATCT and β-actin forward 5′-CCACCATGTACCCAGGCATT-3′ and reverse 5′-CACATCTGCTGGAAGGTGGA-3′.

Each reaction was performed in duplicate using a StepOnePlus™ Real-Time PCR system (4376600, Life Technologies). A no-template control was used along with other samples to check for contamination. The data generated from the real-time PCR was analysed by ANOVA for statistical significance using GraphPad Prism 6.0 software.

### RT-PCR on rib growth plate extract to investigate NMD

Rib cages dissected from 3-week-old mice heterozygous for the MCDS-causing Y632X collagen X mutation were placed in collagenase medium containing 100 (μg/ml) cycloheximide (C7698, Sigma) for 4 h. Growth plates were then dissected form the ribs, placed in 500 μl of TRIzol (15596018, Life Technologies) and homogenized using a microdismembranator as described earlier. RNA was isolated and reverse transcribed to cDNA using the Taqman Reverse Transcription Reagent Kit (N8080234, Applied Biosystems) according to the manufacturer’s protocol. One microlitre of cDNA was PCR amplified using Col10a1 primers flanking the Y632X mutation site: forward 5′-GCCTACGATGTACACGTATG-3′ and reverse 5′-AGGGCTTTAGGATTGCTGAG-3′. PCR products were subjected to restriction enzyme digestion with Nhe1-HF (R3131S, New England Biolabs, Hitchin, SG40TY, UK). Restriction enzyme-digested PCR products (wild-type allele with the size of 553 bp and mutant allele with the sizes of 340 and 213 bp) were separated on 1% agarose gel. The ratio of wt/mutant allele was quantified using ImageJ relative to their levels in the heterozygous genomic DNA. A minus reverse transcriptase (−RT) control containing all the RT-PCR reagents except the reverse transcriptase was set for each sample to check for any possible contamination with genomic DNA.

*Conflict of Interest statement.* None declared.
